# Factors associated with utilization of community health workers in improving access to malaria treatment among children in Kenya

**DOI:** 10.1186/1475-2875-11-248

**Published:** 2012-07-30

**Authors:** James Kisia, Florence Nelima, David Odhiambo Otieno, Kioko Kiilu, Wamalwa Emmanuel, Salim Sohani, Kendra Siekmans, Andrew Nyandigisi, Willis Akhwale

**Affiliations:** 1 Kenya Red Cross Society, P.O Box 40712, Nairobi, 00100, Kenya; 2 University of Nairobi Institute of Tropical and Infectious Diseases, P.O Box 30197, Nairobi, 00100, Kenya; 3 CARE International in Kenya, P.O. Box 43864, Nairobi, 00100, Kenya; 4 Canadian Red Cross, 170 Metcalfe Street, Suite 300, Ottawa, ON, K2P 2P2, Canada; 5 HealthBridge, 1004-1 Nicholas Street, Ottawa, ON, K1N 7B7, Canada; 6 Division of Malaria Control, Ministry of Public Health and Sanitation, Nairobi, Kenya; 7 Department of Disease Prevention and Control, Ministry of Public Health and Sanitation, Nairobi, Kenya

**Keywords:** Malaria, Kenya, Community case management, Community health worker, Children under five

## Abstract

**Background:**

The success of community case management in improving access to effective malaria treatment for young children relies on broad utilization of community health workers (CHWs) to diagnose and treat fever cases. A better understanding of the factors associated with CHW utilization is crucial in informing national malaria control policy and strategy in Kenya. Specifically, little is known in Kenya on the extent to which CHWs are utilized, the characteristics of families who report utilizing CHWs and whether utilization is associated with improved access to prompt and effective malaria treatment. This paper examines factors associated with utilization of CHWs in improving access to malaria treatment among children under five years of age by women caregivers in two malaria endemic districts in Kenya.

**Methods:**

This study was conducted in 113 hard-to-reach and poor villages in Malindi and Lamu districts in the coastal region classified as having endemic transmission of malaria. A cross-sectional household survey was conducted using a standardized malaria indicator questionnaire at baseline (n = 1,187) and one year later at endline assessment (n = 1,374) using two-stage cluster sampling.

**Results:**

There was an increase in reported utilization of CHWs as source of advice/treatment for child fevers from 2% at baseline to 35% at endline, accompanied by a decline in care-seeking from government facilities (from 67% to 48%) and other sources (26% to 2%) including shops. The most poor households and poor households reported higher utilization of CHWs at 39.4% and 37.9% respectively, compared to the least poor households (17.0%). Households in villages with less than 200 households reported higher CHWs utilization as compared to households in villages having >200 households. Prompt access to timely and effective treatment was 5.7 times higher (95% CI 3.4-9.7) when CHWs were the source of care sought. Adherence was high regardless of whether source was CHWs (73.1%) or public health facility (66.7%).

**Conclusions:**

The potential for utilization of CHWs in improving access to malaria treatment at the community level is promising. This will not only enhance access to treatment by the poorest households but also provide early and appropriate treatment to vulnerable individuals, especially those living in hard to reach areas.

## Background

Malaria is a problem of public health importance in Kenya where it is estimated to cause 20% of all deaths of children below five years of age [1]. Community case management of malaria (CCM) is one of the new approaches adopted by malaria endemic countries to reduce the burden of malaria for vulnerable populations. According to the World Health Organization (WHO), CCM is based on the evidence that well-trained and supervised community health workers can provide prompt and adequate treatment to fever cases within 24 hours to help reduce morbidity and mortalities associated with malaria among under-five children in Africa [2].

Community health workers (CHWs) are in level one of the Kenyan healthcare service provision and a central pillar of primary health care delivery at the community level. Furthermore, Kenya’s Community Health Strategy 2006 highlights the provision of preventive health services by CHWs and clearly provides structures that operationalize service provision at community level [3]. CHWs provide the link between the health facilities and community members and are given responsibility for health promotion, disease prevention as well as treatment of specific diseases, such as uncomplicated malaria [4].

There has been growing evidence on the effectiveness of CCM globally [5-8]. This evidence suggests that access to effective malaria treatment improves when it is provided by CHWs, particularly in areas where families face barriers to accessing health facilities. In Kenya, multiple factors related to affordability, acceptability and availability interact to influence level of access to prompt and effective malaria treatment among the poor [9]. CCM delivered by CHWs has the potential to reduce such barriers through reduced costs associated with fever treatment (free drugs, no transport required, low opportunity cost), interaction with a fellow community member who understands the family’s situation, availability of care givers outside normal “business hours” and more frequent follow-up.

Evidence that the success of CCM in improving access to effective malaria treatment relies on broad utilization of CHWs is best illustrated in a recent study conducted in Uganda, which found that despite the fact that 42% of households had access to a community-based distributor of the recommended anti-malarial drug, only 25% of cases used the drug [10]. Some of the key characteristics that have been shown to be associated with health care-seeking practices and that may influence the utilization of CHWs in community settings include geographic access to health facilities and socioeconomic status of households [11].

As part of an evaluation of a pilot CCM project in Kenya, this study examines the extent to which CHWs were utilized, the characteristics of the families who reported utilizing CHWs and whether utilization was associated with improved access to prompt and effective malaria treatment. A better understanding of the factors associated with CHW utilization is expected to help inform national malaria control policy and strategy in Kenya.

## Methods

### Study context

The study was conducted in Malindi and Lamu districts in the Coast Province of Kenya. The region is characterized by a low level of access to health facilities for much of the population due to difficult geographical barriers, semi-arid climate and prevailing high level of poverty. Many villages are located far from main access roads with terrain that is impassable by motor vehicles, including some areas located across rivers or swamps. Lamu district includes several islands. During the rainy season, widespread flooding and wild animals from neighboring game parks further aggravate inaccessibility. At the time of the project design, Coast province was classified as a high malaria transmission zone; however, more recent Kenya Malaria Indicator surveys [1,12] suggest that concerted malaria prevention and control programmes are bearing fruits and transmission is declining over time.

The pilot CCM project was implemented by Kenya Red Cross Society (KRCS) that works with an extensive network of community-based volunteers in these two districts. The project was funded by the Canadian International Development Agency (CIDA) and technical support was provided by WHO and the Division of Malaria Control (DOMC, Ministry of Public Health and Sanitation - MoPHS). The pilot phase had an estimated target population of 9,600 children 3–59 months of age living in 113 communities (total population 71,850) that were generally considered “hard to reach”.

The objective of the pilot project was to increase access to artemisinin-based combination therapy (ACT) as malaria treatment for children under five in hard to reach areas. The project’s management structure was set up in such a way as to strengthen the capacity of the health system in these districts in accordance with the national Community Health Strategy, being fully integrated within the roles and responsibilities of existing health service providers. For instance, the district malaria focal person worked in close collaboration with the KRCS Project Officer to manage the project activities and Community Health Extension Workers (CHEWs), who are full-time employees of the MoPHS based in health facilities in the targeted areas, functioned as coaches to selected groups of CHWs. ACT supplies (artemether-lumefantrine (AL) packs) were supplied through normal MoPHS channels where Kenya Medical Supplies Agency provided facilities with medication based on needs (*i.e.* the District Pharmacy) with additional logistic support from KRCS.

In January 2009, 113 CHWs (one per village) were selected by Village Committees, based on the government’s Community Health Strategy 2006 criteria, which required them to be resident in the community, respected members of the community and literate. Preference was given to pre-existing KRCS volunteers in the village while the project recruited new CHWs and registered them as new volunteers in areas without KRCS volunteers. Project induction training was conducted by DOMC. Further refresher training for CHWs was done after six months. These trainings were conducted using approved training curriculum whose content incorporated Integrated Management of Childhood Illness (IMCI) materials that covered important and relevant components like signs and symptoms of uncomplicated malaria, danger signs for referral, correct dispensing of AL (dose regimen, importance of compliance, potential adverse reactions), the referral process, record keeping and community health promotion topics, *e.g.* malaria prevention through use of insecticide-treated nets (ITN) and intermittent preventive treatment in pregnancy (IPTp).

At the time of the project design, the WHO and DoMC guidelines for treatment of fever in children under five did not require diagnosis prior to treatment. Rapid diagnostic tests for malaria were not regularly available at a significant proportion of health facilities due to cost constraints. Therefore, the use of RDTs was not included in the project. In order to prescribe the AL at the community level by CHWs, the project applied for and received a formal waiver for prescription of AL across the counter from the Pharmacy and Poisons Board together with DOMC. Supplies of age-appropriate AL packs were distributed to CHWs by their respective coaches on a monthly basis, based on supplies used and monitoring reports of child fever cases treated. Compliance was measured from the volunteer recording forms and discussion with care givers. Referrals to a health facility were done when there were danger signs (*e.g.* complicated malaria case), adverse drug reactions or treatment failure.

Close supervision and monitoring of CHW activities was done throughout the project implementation period. CHEWs met every two weeks with CHWs at a designated health facility to verify the CHW monitoring reports and address any concerns. The health facility nurse often participated in these meetings and this contributed to enhanced coordination, particularly for referrals. The project was also discussed at monthly district level meetings and independently monitored by provincial health management team members.

### Project evaluation methods

An evaluation of changes occurring during the project period was conducted by carrying out a household survey in the targeted communities before and after the intervention. Baseline and endline surveys were conducted in December 2008 and December 2009, respectively. Two-stage cluster sampling was used. In the first stage, 30 clusters (defined as villages) were randomly selected using probability proportional to size. In the second stage, 30 households were randomly selected from a list of all households in the cluster with at least one child 3–59 months of age.

Two semi-structured survey questionnaires were administered, both based in large part on standardized Malaria Indicator Survey (MIS) modules [13]. Data was collected on the household member list, household characteristics, mosquito net inventory, reproductive history of the woman caregiver, fever and health seeking behaviours in the youngest and next-to-youngest children, and knowledge of AL and malaria. A series of questions was added for the endline survey to assess the frequency and nature of the household’s interaction with CHWs. Two interviewers were assigned to collect data from each sampled household; one interviewed the head of the household (Form A) and the other the selected woman caregiver over 15 years of age (Form B).

Training of interviewers for field data collection procedures was conducted using KMIS 2007 materials and manuals. During data collection, field supervisors undertook daily spot checks including checking 10% of the completed questionnaires to confirm that the interviews took place appropriately. During the spot checks, at least three households were re-interviewed by the field supervisor to verify the information collected and any discrepancies were corrected before data entry in Microsoft Access.

### Statistical data analysis

The main outcome variables of this study were malaria care-seeking and treatment practices. Data on child fever treatment practices was taken from the module administered to women caregivers of children 3–59 months. In households with data for two children, one child was randomly selected. From this sample of one child per household, children with fever in the last two weeks were selected for inclusion in the sub-sample to assess fever treatment practices.

Variables related to treatment seeking practices, in particular the source of advice/treatment sought and associated actions, were analysed by household characteristics including caregiver age and education level, household wealth rank (see below) and size, attendance to antenatal clinic (ANC) as a proxy for access to routine health services, malaria-related knowledge and practices (IPTp coverage, knowledge of AL as the recommended anti-malarial drug, knowledge of sleeping under bed net to prevent malaria) and village size. Source of treatment was categorized as CHW and Others, which included public and private health sector sources. Treatment was considered prompt when it was given within 24 hours of fever onset. Effective treatment was defined as receiving AL, the recommended first-line anti-malarial.

Household-level variables on asset ownership, water source, toilet facility and house flooring material were used to create a wealth index using Principal Component Analysis, a methodology that is commonly applied to DHS datasets [14]. Each household was given a factor score, which was used to assign a rank. In order to heighten the sensitivity of the wealth index to the lowest and highest groups, a three-level ranking was created, with the lowest rank including the poorest 20% of households, the middle rank the central 60% and the highest rank the least poor 20% of households. Internal consistency of the wealth index was assessed. In both surveys, the poorest 20% of households all shared common characteristics – all relied on surface water sources, none of them reported using toilet facilities (all used field/bush) and none of them reported ownership of any assets (with the exception at baseline of some households owning a bicycle). The least poor 20% of households were most likely to use improved sources of water, use improved toilet facilities and own the assets under question. These results suggest that the wealth rank variable generally performed as desired and may assist in understanding the differences between households associated with relative wealth, even though it is clear that, in general, all households in the project area were disadvantaged socioeconomically.

One CHW was assigned to each village regardless of the size of that village. The size of villages targeted by the project ranged from a population of 107 to 3,173 (mean 636, median 500), approximately 23 to 690 households. Village size was recoded into a four-level categorical variable: <60, 61 to 100, 101 to 200, and >200 households.

Pearson’s chi-square test for categorical variables was used to test for differences across groups; tests associated with a p-value less than 0.05 were considered as evidence for a significant difference. Data were analysed using Stata version 11.2.

## Ethical approval

Ethical clearance for this study was granted by the Kenyatta National Hospital’s Research and Ethics Committee, Ministry of Health, Kenya. Within limits of the project capability, all study participants who were considered ill were either treated or referred to the nearest health facility. Voluntary participation and confidentiality of information was ensured and informed consent secured.

## Results

Data was available for 770 households at baseline (86% response rate) and 861 households at endline (96% response rate). Fever prevalence in the past two weeks among the full sample of children 3–59 months was similar in both surveys: 36.9% at baseline (N = 1187) and 39.8% at endline (N = 1374; p = 0.14). The remainder of the analysis is based on a randomly selected sub-sample of children (one per household) who reported having fever in the past two weeks. Characteristics of the sub-sample were similar to those of the full sample (see Additional file 1).

Table 1 presents a description of the sample characteristics at baseline and endline. Woman caregiver respondent characteristics were similar in both samples. The mean age of women caregivers was 33 years in both surveys, with low literacy levels and over half reporting they had no formal education. Household characteristics were largely similar as well, although the endline sample households reported having fewer assets (*e.g.* radio, bicycle) and more bed nets. The size of villages in which the household lived was equally distributed for both surveys.

**Table 1:** Sample household, caregiver and community characteristics

**Characteristic**	**BASELINE N = 269**	**ENDLINE N = 345**	**p-Values**
Women caregiver education level			0.125
None	53.2 (143)	57.7 (199)	
Primary	43.5 (117)	41.2 (142)	
Secondary	3.4 (9)	1.2 (4)	
Woman caregiver age category			0.332
<=20 y	21.2 (57)	15.7 (54)	
21-30 y	44.6 (120)	47.1 (162)	
31-50 y	26.7 (72)	29.4 (101)	
51+ y	0	1.1 (4)	
Unknown	7.4 (20)	6.7 (23)	
Male household head	84.1 (216)	81.7 (282)	0.285
Household owns radio	40.9 (105)	32.8 (113)	0.041
Household owns bicycle	56.4 (145)	48.1 (166)	0.044
Household owns mosquito nets	81.3 (209)	89.3 (308)	0.006
Village size			0.531
≤ 60 households	21.9 (59)	18.6 (64)	
61 to 100 households	24.5 (66)	27.8 (96)	
101 to 200 households	31.6 (85)	32.2 (111)	
>200 households	21.9 (59)	21.5 (74)	
Household wealth rank			0.335
Most poor	22.3 (60)	23.8 (82)	
Poor	54.3 (146)	57.7 (199)	
Least poor	23.4 (63)	18.6 (64)	

A comparison of the source of advice/treatment sought by caregivers for their child’s fever is shown in Table 2. At baseline, over two thirds (67%) of women caregivers reported seeking treatment for their child’s fever from government facilities, while a third sought treatment from other sources (25% of these sought health care from shop keepers). However, the situation reversed at endline whereby 34.6% of caregivers reported seeking advice/treatment from CHWs, a significant increase from 2% at baseline. This was accompanied by a decline in advice/treatment seeking from government facilities, which dropped from 67% to 48% and “other” sources which decreased from 26% to 2%.

**Table 2:** Source of treatment for children with fever in past two weeks among those who reported seeking treatment

**Source of advice/treatment**	**Baseline (N = 235)**		**Endline (N = 298)**	
	**n**	**%**	**n**	**%**
CHW/Red Cross volunteer	5	2.1	103	34.6
Government health facilities	157	66.8	143	48.0
Private medical sector	12	5.1	46	15.4
Other sources	59	26.0	6	2.0

From Table 3, women caregiver characteristics including education level, age group, ANC attendance and IPT during last pregnancy were not significantly associated with use of CHW services for child fever advice/treatment. However, women who knew that AL was the new anti-malaria drug were also more likely to have sought advice/treatment from a CHW than women who did not know. The most poor households and poor households showed higher proportions (39.4% and 37.9%, respectively) of utilization of CHWs compared to the least poor households (17.0%). Households located in villages with less than 200 households were more likely to utilize CHWs compared to households in villages with more than 200 households, where utilization of CHW services was lowest (16.9%).

**Table 3:** Women caregiver, household and village characteristics associated with utilization of CHW services for child fever treatment

**Characteristic**	**N**	**CHW % (n)**	**Other % (n)**	**p-value**
Overall	298	34.6 (103)	65.4 (195)	
Women caregiver education				
No formal education	169	34.9 (59)	65.1 (110)	0.885
Primary/secondary	129	34.1 (44)	65.9 (85)	
Women caregiver age group				
≤20 y	47	34.0 (16)	66.0 (31)	0.993
21-30 y	136	36.0 (49)	64.0 (87)	
31-40 y	81	35.8 (29)	64.2 (52)	
41+ y	12	33.3 (4)	66.7 (8)	
Attended ANC during last pregnancy				
Yes	196	35.2 (69)	64.8 (127)	0.581
No	97	32.0 (31)	68.0 (66)	
IPT (2+ doses SP) during last pregnancy				
Yes	140	32.9 (46)	67.1 (94)	0.674
No	27	37.0 (10)	63.0 (17)	
Knowledge of AL as new anti-malarial drug				
Yes	104	44.2 (46)	55.8 (58)	0.010
No/Don’t know	194	29.4 (57)	70.6 (137)	
Identified sleeping under net as way to prevent malaria				
Yes	239	36.4 (87)	63.6 (152)	0.179
No	59	27.1 (16)	72.9 (43)	
Household wealth rank				
Most poor	71	39.4 (28)	60.6 (43)	0.012
Poor	174	37.9 (66)	62.1 (108)	
Least poor	53	17.0 (9)	83.0 (44)	
Household size				
2 to 4	71	32.4 (23)	67.6 (48)	0.425
5 to 7	132	31.8 (42)	68.2 (90)	
8 or more	93	39.8 (37)	60.2 (56)	
Village size				
<60 households	57	40.4 (23)	59.7 (34)	0.008
61-100 households	82	41.6 (34)	58.5 (48)	
101-200 households	94	37.2 (35)	62.8 (59)	
>200 households	65	16.9 (11)	83.1 (54)	
Visited by CHW in past 3 months				
Yes	153	63.4 (97)	36.6 (56)	<0.001
No	176	39.2 (69)	60.8 (107)	

Table 4 shows the results of the analysis of the treatment status for children with fever according to the source of treatment sought by the caregiver. When CHWs were utilized, the child’s odds of receiving AL within 24 hours were 5.7 times higher (95% CI 3.4, 9.7) compared to other sources of treatment. Similar results were observed for treatment within 48 hours or at any time following the onset of the fever. Among children treated with AL, adherence (taking AL for three days) was high regardless of whether treatment was sought from a CHW (73.1%) or public health facility (66.7%).

**Table 4:** Timing of provision of AL by source of the treatment

**Timing of AL treatment**	**Source of advice/treatment**		**p-value**
	**CHW**	**Other**	
AL given within 24 hours	57.3 (59)	19.0 (37)	<0.001
AL given within 48 hours	79.6 (82)	36.4 (71)	<0.001
AL given at any time	90.3 (93)	45.1 (88)	<0.001

## Discussion

This study has demonstrated the extent to which CHWs were utilized by caregivers of children under five years old in treatment of fevers. In addition to this, the results showed the characteristics of families who reported utilizing CHWs in this study and the association between utilization of trained and supervised CHWs and improved access to prompt and effective malaria treatment in Kenya.

This study’s results support the possibility that CHWs can be influential in changing the health seeking behavior of families in hard to reach villages in developing countries. An increase was observed in caregivers seeking advice/treatment from recommended sources (including CHWs/KRCS volunteers) as opposed to shop-keepers who were not a source of effective treatment of malaria (AL) in this context at the time of the study. One possible explanation for this increase is that CHWs were more likely to be closer to the families needing care than the shopkeepers. Furthermore, the CHWs provided free malaria treatment after fever assessment compared to the shopkeepers who sold the medications without further diagnosis and medication services. In addition to this, the increased and sustained level of awareness and response to the new malaria treatment being promoted through awareness campaigns by various groups (*e.g.* community health committees, mass media programme for affordable facility malaria programme), may have also been contributing factors.

CHWs’ in-depth understanding of the community’s values, their residency within the community, the training and health- related equipment provided by KRCS likely enabled them to continuously cultivate good client-service provider relationships compared to shopkeepers and health facility staff. Some of these factors may encourage the women with children under five years old to seek prompt and timely care. Such findings have also been reported by studies conducted in developing countries such as Uganda [10,15] and Mali [6], which share many demographic characteristics with Kenya.

Some reduction was observed in caregivers seeking advice/treatment from government health facilities at the end of the project, concurrent with an increased preference for CHWs’ advice/treatment. Given the cross-sectional nature of the survey, it was not possible to determine the reasons for this change. However, previous research in Kenya has documented various barriers on both the supply and demand side to prompt and effective treatment for malaria in children under five years of age at government health facilities [9] and these factors may have played a role in this context. One potential positive result of this shift is a decrease in the health facility caseload of uncomplicated child fever cases. This has been documented by Tiono *et al.*[6] in a rural district of Burkina Faso who observed that community case management of child fever cases freed up health facility staff to treat a higher proportion of other illness.

Furthermore, it has been observed in other published studies that trained and supervised CHWs enhanced malaria treatment adherence that enabled the medication to clear malaria parasites from the client’s blood and was more cost effective compared to health facility-based care [16].

Results showed that community size and household wealth rank were important characteristics influencing the utilization of CHW services. The project allocated one CHW per village (ranging from a 23–690 households) while the Community Health Strategy (2006) recommended one CHW per 100 persons in Coast province, a region with a population density ranging from 37–39 persons/km^2^ (Community Strategy 2006). Our findings revealed a higher proportion of families seeking advice/treatment from CHWs working with less than 200 households, suggesting that CHWs are more highly utilized when allocated fewer households. This is consistent with the current guidelines articulated within Kenya’s Community Health Strategy.

While provision of care was not based on household wealth ranking, this study demonstrated that mothers from the poorest households were more likely to seek advice/treatment from CHWs. Possible reasons for this included the fact that community case management was offered for free, CHWs provided prompt treatment at the household level and there was high confidence in CHWs whom communities nicknamed as “doctors of one drug” and “malaria doctor”. A study conducted in rural Bangladesh, Asia by Ahmed *et al.*[17] comparing health seeking behavior of elderly *versus* younger household members found insignificant difference between these two groups in terms of health seeking behavior but noted high consultation of CHWs services; poverty was reported as the most important determinant of health seeking behavior. These findings are in agreement with the current study findings that the poorest were more likely to seek CHWs services for community case management of malaria.

One goal in malarial control is to ensure access to prompt and effective treatment among young children. The findings from this study demonstrate that access to the recommended first-line anti-malarial treatment for uncomplicated malaria was dramatically improved by use of CHWs who were trained to provide presumptive treatment of fever cases with AL medication. This corresponds well with other studies conducted in Zambia, Zaire and Asia where CHWs were effective in rapid diagnosis and treatment of malaria [18-20].

Even though this study’s findings corroborated other studies conducted in developing countries regarding successes learnt from use of trained and supervised CHWs in community case management, the cost of employing the service of the recommended number of CHWs were too prohibitive to our study hence it could not effectively assess the realistic impact of the Community Health Strategy 2006’s recommendations on this. In addition to this, there was no control group to not only assess internal validity but also the generalizability of its findings while effectively controlling for the effects of secular change, bias and confounders.

## Conclusions

The results of this study provide evidence that use of trained and supervised community health workers in community case management improved management of uncomplicated child fever cases in hard to reach villages in Malindi and Lamu District in Coastal Province of Kenya. In addition to this, poverty seems to be closely linked to child caregivers seeking services of community-based service providers, highlighting the impediment of poverty towards accessibility of cost sharing services widely practiced in Kenyan public health facilities. Policy actions to address barriers to effective utilization of CHWs in healthcare delivery should be scaled up in such hard to reach communities. The government and partners should, therefore, invest more in mechanisms which support CHW utilization especially the roll out of the Community Health Strategy 2006 as part of successful control of malaria and other infectious diseases.

## Competing interests

The authors declare that they have no competing interests.

## Authors’ contributions

JK, WA and SS designed the pilot project; JK, SS, WE and AN contributed to the project implementation; KK, DO, SS, FN, WE, AN and KS participated in the analysis and interpretation of the study results; FN wrote the first draft of the manuscript and all authors reviewed and approved the final draft.

## Supplementary Material

Additional file 1 Sample household, caregiver and community characteristics. Click here for file
