# Supplementary material for: Factors associated with utilization of community health workers in improving access to malaria treatment among children in Kenya
Source: Malar J. 2012 Jul 30;11:248. doi: 10.1186/1475-2875-11-248 (PMC3473249; doi:10.1186/1475-2875-11-248)
Supplement: Additional file 1 — Sample household, caregiver and community characteristics. [file 1475-2875-11-248-S1.doc]

**Additional file 1** Sample household, caregiver and community characteristics

|  | **Overall Sample**  **Baseline End-line p**  **% (n) % (n)** | **Children with fever sub-sample**  **Baseline End-line p**  **% (n) % (n)** |
| --- | --- | --- |
| **N**  Woman caregiver education level  None  Primary  Secondary  Woman caregiver age category  <=20 y  21-30 y  31-50 y  51 + y  Unknown  Male household head  Household owns radio  Household owns bicycle  Household owns mosquito nets  Household wealth rank  Poorest    Poor  Least poor | **740**  **856**  0.42  54.7(417) 57.8(497)  42.3(323) 39.7(341)  3.0(23) 2.6(22)  0.87  14.4(110) 13.1(113)  47.7(364) 47.4(408)  29.9(228) 31.7(273)  1.3(10) 1.5(13)  6.7(51) 6.2(53)  81.1(600) 81.7(699) 0.77    39.3(291) 35.5(304) 0.12  55.3(409) 47.3(405) 0.002  81.4(602) 88.0(753) <0.001  0.93  23.0(170) 22.3(191)  56.6(419) 56.7(485)  20.4(151) 21.0(180) | **269**  345  0.13  53.2(143) 57.7(199)  43.5(117) 41.2(142)  3.4(9) 1.2(4)  0.33  21.2(57) 15.7(54)  44.6(120) 47.1(162)  26.7(72) 29.4(101)  1.1(4)  7.4(20) 6.7(23)  84.1(216) 81.7(282) 0.29  40.9(105) 32.8(113) 0.04  56.4(145) 48.1(166) 0.04  81.3(209) 89.3(308) 0.006  0.34  22.3(60) 23.8(82)  54.3(146) 57.7(199)  23.4(63) 18.6(64) |
